# Investigation of the quantification of hemoglobin and cytochrome-c-oxidase in the exposed cortex with near-infrared hyperspectral imaging: a simulation study

**DOI:** 10.1117/1.JBO.25.4.046001

**Published:** 2020-04-01

**Authors:** Luca Giannoni, Frédéric Lange, Ilias Tachtsidis

**Affiliations:** University College London, Department of Medical Physics and Biomedical Engineering, London, United Kingdom

**Keywords:** biomedical optics, hyperspectral imaging, Monte Carlo methods, brain metabolism, brain hemodynamics and oxygenation, cytochrome-c-oxidase

## Abstract

**Significance:** We present a Monte Carlo (MC) computational framework that simulates near-infrared (NIR) hyperspectral imaging (HSI) aimed at assisting quantification of the *in vivo* hemodynamic and metabolic states of the exposed cerebral cortex in small animal experiments. This can be done by targeting the NIR spectral signatures of oxygenated (${\mathrm{HbO}}_{2}$) and deoxygenated (HHb) hemoglobin for hemodynamics as well as the oxidative state of cytochrome-c-oxidase (oxCCO) for measuring tissue metabolism.

**Aim:** The aim of this work is to investigate the performances of HSI for this specific application as well as to assess key factors for the future design and operation of a benchtop system.

**Approach:** The MC framework, based on Mesh-based Monte Carlo (MMC), reproduces a section of the exposed cortex of a mouse from an *in vivo* image and replicates hyperspectral illumination and detection at multiple NIR wavelengths (up to 121).

**Results:** The results demonstrate: (1) the fitness of the MC framework to correctly simulate hyperspectral data acquisition; (2) the capability of HSI to reconstruct spatial changes in the concentrations of ${\mathrm{HbO}}_{2}$, HHb, and oxCCO during a simulated hypoxic condition; (3) that eight optimally selected wavelengths between 780 and 900 nm provide minimal differences in the accuracy of the hyperspectral results, compared to the “gold standard” of 121 wavelengths; and (4) the possibility to mitigate partial pathlength effects in the reconstructed data and to enhance quantification of the hemodynamic and metabolic responses.

**Conclusions:** The MC framework is proved to be a flexible and useful tool for simulating HSI also for different applications and targets.

## 1 Introduction

Hyperspectral imaging (HSI) is an emerging optical technique for biomedical applications that can be potentially used to quantitatively monitor *in vivo* changes in the metabolic and hemodynamic states of the brain, specifically on the exposed cortex. HSI provides extensive spectral information, in addition to spatial data, by acquiring images over a broad range of the light spectrum at numerous and contiguous wavelength bands.^1^^,^^2^ Changes in the concentrations of relevant biomarkers, such as oxyhemoglobin (${\mathrm{HbO}}_{2}$) and deoxyhemoglobin (HHb), can be retrieved by measuring the intensity changes of multiple different wavelengths of reflected light after having interacted with the cerebral tissue. These light intensity changes originate from variations in the optical properties of brain tissue during physiological processes, e.g., changes in brain oxygenation and perfusion.^3^^,^^4^ HSI can also be used to target the changes of the redox state of cytochrome-c-oxidase (CCO), which is a chromophore directly involved in the production of adenosine triphosphate in the mitochondria.^5^ CCO has a high specificity as a biomarker for monitoring brain metabolism, due to its high concentration in the cortical tissue.^5^^,^^6^

Metabolic monitoring through CCO is primarily performed noninvasively via broadband near-infrared spectroscopy (bNIRS), which, similar to HSI, analyzes spectroscopically a large number of wavelengths (tens to hundreds) in the near-infrared (NIR) range between 780 and 900 nm.^6^ This specific range is chosen due to the presence of a predominant broad peak in the absorption spectrum of the copper CuA redox center of CCO, which enables a better differentiation of the CCO signal from those of ${\mathrm{HbO}}_{2}$ and HHb.^6^^,^^7^ However, bNIRS is not a wide-field imaging technique and it is limited in terms of spatial resolution, due to the high-scattering properties of biological tissue in the NIR range. Furthermore, bNIRS only provides information about changes in metabolism and hemodynamics that are averaged over relatively large volumes of tissue (typically from 1 to $100\;{\mathrm{cm}}^{3}$), which can include both blood vessels as well as surrounding extravascular tissue, since it is based on measuring NIR light diffusing through the scalp, the skull, and the gray and white matter. For this reason, looking directly at the exposed cerebral cortex using HSI in the NIR range could provide additional and more exhaustive information about brain metabolism and hemodynamics, in particular by spatially differentiating between the regions where the two processes are primarily located, i.e., the pial vasculature and the surrounding subpial cortical tissue, for the hemodynamic response and the metabolic response, respectively. The NIR hyperspectral approach targeting the exposed cortex can thus be used to obtain a deeper understanding of brain physiology during different conditions, such as hypoxic-ischemia or other similar abnormal alterations in brain oxygenation.

We present here a Monte Carlo (MC) framework that simulates NIR HSI of the hemodynamic and metabolic states of the exposed cortex. To our knowledge, no MC computational analysis has been published before to reproduce wide-field HSI simultaneously targeting the changes in ${\mathrm{HbO}}_{2}$, HHb, and the oxidative state of CCO (oxCCO). This approach is used to investigate the feasibility and performances of using HSI in the NIR range to quantitatively measure changes in concentrations of the abovementioned biomarkers, by simulating a realistic portion of mouse brain cortex (created from an *in vivo* image) during changes from cerebral normoxia to acute hypoxia. In particular, the computational analysis focuses on: (1) assessing the capacity of HSI to reconstruct spatial maps of metabolic and hemodynamic activity; (2) evaluating the accuracy of HSI in quantitatively estimating relative changes in the concentrations of ${\mathrm{HbO}}_{2}$, HHb and oxCCO; (3) investigating what is the optimal selection and number of wavelength bands to use for HSI to simultaneously image ${\mathrm{HbO}}_{2}$, HHb, and oxCCO; and (4) studying the effects, influence, and magnitude of cross talk and partial pathlength effects affecting the hemoglobin and oxCCO signals. We define cross talk as the erroneous measured change in the concentration of a chromophore that is induced by the genuine concentration change of another chromophore.^6^ Conversely, we define the partial pathlength effect as the erroneous measured change in a chromophore concentration due to large variance in the photon pathlengths or to incorrect estimates of the latter.^6^^,^^8^^,^^9^

Finally, an alternative hyperspectral illumination and detection configuration, as well as different data processing methods, are also explored and tested to find which could be the ideal HSI methodology to efficiently and reliably monitor hemodynamics and metabolism in the exposed cortex. This last aspect is significant in the context of designing and operating an HSI benchtop system that can experimentally achieve the same level of performances *in vivo* on small animal models, such as mice and rats.

## 2 Methods

The Monte Carlo HSI framework has been developed using mesh-based Monte Carlo (MMC) and iso2mesh packages. MMC, is an open-source MC solver for photon migration in three-dimensional (3-D) turbid media, originally developed by Fang et al.^10^^–^^13^ Differently from other existing MC software packages, either designed for layered (such as Monte Carlo multilayered^14^) or voxel-based media (e.g., Monte Carlo eXtreme^15^ and tMCimg^16^), MMC can represent a complex domain using a volumetric mesh with triangular surfaces. This modeling technique greatly improves the accuracy of the solutions when modeling objects with curved and complex boundaries, as well as providing an efficient way to sample the problem domain. Thanks to that and to the use of a fast-ray tracing algorithm using Plücker coordinates for rapidly calculating tetrahedron intersections, MMC is also able to efficiently speed up computational time and use less memory during the simulation.^10^ MMC is coupled with a mesh-generation and processing toolbox called iso2mesh,^17^^,^^18^ used to create a volumetric meshed domain that replicates the geometry and structure of cerebral tissue and vasculature from a two-dimensional (2-D) *in vivo* image of the exposed cortex. Finally, recent releases of the MMC package have implemented the capability to also simulate arbitrary wide-field sources and detectors over large surface areas using mesh retessellation algorithms with high computational efficiency.^12^^,^^19^ This aspect is crucial for the simulation of HSI, due to the requirement of accurate and reliable representation of 2-D illumination and detection patterns that are characteristic of this optical imaging technique.

### 2.1 Geometry and Optical Properties of the Domain

**Fig. 1:**
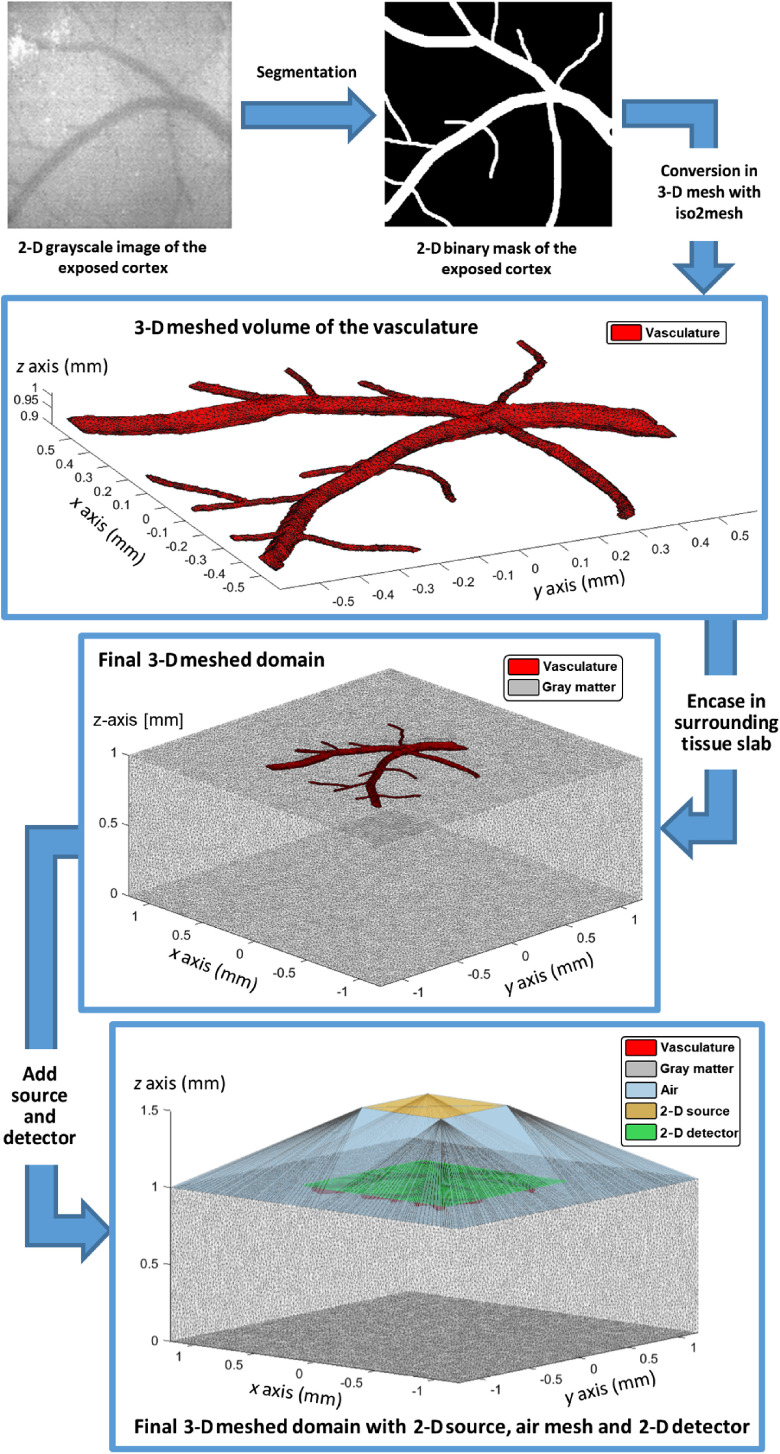
Workflow diagram of the methodology used in the Monte Carlo HSI framework to create a 3-D meshed domain of the exposed cortex: from an *in vivo* 2-D image (in grayscale), a binary mask is first created (in black and white) identifying the two media; then a 3-D mesh of the pial vasculature (in red) is generated, as well as a slab of subpial gray matter (in gray) encasing it; finally a 2-D source (in gold) and a 2-D detector (in green) are added to the final domain, with an additional mesh made of air (in cyan) filling the gap between the source and the cortex mesh.

The MC framework implements a methodology to produce a realistic tetrahedral-mesh heterogeneous domain of a section of the exposed cerebral cortex of a mouse (including pial vasculature and subpial brain tissue) from a 2-D grayscale image acquired *in vivo* using a conventional charge-coupled device. The workflow diagram describing this methodology is illustrated in Fig. 1.

The grayscale image of the exposed cortex, showing a 1.2×1.2  mm field of view (FOV) of the surface of the brain of a mouse and composed of 400×400  pixels, is first manually segmented to obtain a binary mask that differentiates between blood vessels and the surrounding brain tissue. A 3-D binary volume of the pial vasculature (1.2×1.2×0.1  mm) is then generated by expanding the mask along the vertical direction while symmetrically eroding the sections of the vessels from the central plane. This is done to replicate the curvature of the vascular geometry. The 3-D binary volume of the pial vasculature is then converted into a meshed volume using iso2mesh, constituting the first medium of the final domain. The pial vasculature volume is the encased in a 2.4×2.4×1  mm slab reproducing the surrounding mouse subpial gray matter. The extra layers added to the 1.2×1.2  mm FOV have the purpose of minimising boundary effects during the MC simulations.

Both media in the domain are defined by their geometry as well as by the associated optical properties (absorption coefficient $\mu_{a}$, scattering coefficient $\mu_{s}$, anisotropy g, and refractive index n). The medium that replicates the mouse subpial gray matter is considered to be made of water ($H_{2}O$), lipid (fat), different concentrations of ${\mathrm{HbO}}_{2}$ and HHb (according to the fraction of blood and oxygen saturation level in the tissue), and different concentrations of the redox states of CCO, namely oxCCO and reduced CCO (redCCO). The medium reproducing both major and minor pial vessels (about 100 and 20  μm in diameter, respectively) includes water, fat, as well as ${\mathrm{HbO}}_{2}$ and HHb in different concentrations, according to the oxygen saturation value selected for the pial vasculature.

The composition and the optical properties of the two media are based on equations and reference data by Jacques.^20^ Standard values, characteristic of general biological tissues, are assumed for the anisotropy and the refractive index of all the media of the domain, setting g equal to 0.9 and n equal to 1.365.^21^ The scattering coefficient $\mu_{s}(\lambda )$ is considered to be dependent only on the given wavelength λ of the incident photon packet.^20^ The absorption coefficient $\mu_{a}(\lambda )$ of each medium of the simulated domain is estimated as the sum of the single absorption coefficients, $\mu_{a,H_{2}O}(\lambda )$, $\mu_{a,\text{fat}}(\lambda )$, $\mu_{a,{\mathrm{HbO}}_{2}}(\lambda )$, $\mu_{a,\mathrm{HHb}}(\lambda )$, $\mu_{a,\mathrm{oxCCO}}(\lambda )$, and $\mu_{a,\mathrm{redCCO}}(\lambda )$, at the given wavelength λ, of the major chromophores composing the medium, i.e., water, fat, ${\mathrm{HbO}}_{2}$, HHb, oxCCO, and redCCO, respectively, and weighted accordingly to their content in it.^20^ The data for $\mu_{a,H2O}(\lambda )$ and $\mu_{a,\text{fat}}(\lambda )$ in the NIR range are taken from Matcher et al.,^22^ for water, and van Veen et al.,^23^ for fat (Table 4 in Appendix). The values of $\mu_{a,{\mathrm{HbO}}_{2}}(\lambda )$, $\mu_{a,\mathrm{HHb}}(\lambda )$, $\mu_{a,\mathrm{oxCCO}}(\lambda )$, and $\mu_{a,\mathrm{redCCO}}(\lambda )$ are calculated from the molar extinction coefficients $\varepsilon_{{\mathrm{HbO}}_{2}}(\lambda )$, $\varepsilon_{\mathrm{HHb}}(\lambda )$, $\varepsilon_{\mathrm{oxCCO}}(\lambda )$, and $\varepsilon_{\mathrm{redCCO}}(\lambda )$ of ${\mathrm{HbO}}_{2}$, HHb, oxCCO, and redCCO, respectively. In particular, for the oxCCO and redCCO contributions, this is done according to their selected concentrations [oxCCO] and [redCCO] in the given medium, whereas for the contributions of ${\mathrm{HbO}}_{2}$ and HHb, the average molar concentration of hemoglobin [Hb] in blood, the content B of blood in the specific medium and the oxygen saturation S are taken into account.^20^ The molar extinction coefficients $\varepsilon_{{\mathrm{HbO}}_{2}}(\lambda )$ and $\varepsilon_{\mathrm{HHb}}(\lambda )$ of ${\mathrm{HbO}}_{2}$ and HHb are taken from Matcher et al.,^24^ whereas the molar extinction coefficients $\varepsilon_{\mathrm{oxCCO}}(\lambda )$ and $\varepsilon_{\mathrm{redCCO}}(\lambda )$ of oxCCO and redCCO were measured by John Moody at the University of Plymouth in the bovine heart^6^ (Table 4 in Appendix).

The meshed domain of a section of mouse brain cortex is then integrated with a wide-field planar source for hyperspectral illumination at numerous wavelengths. The 2-D source has dimensions equal to 0.6×0.6  mm and is centered on the slab. It is also parallel to the top surface of the meshed domain, at a distance from it equal to 0.5 mm. The photon packets at each given wavelength λ are launched from the surface of the planar source and evenly distributed over a 1.2×1.2  mm central section of the top surface of the domain, with a beam divergence of 90 deg. The MMC package implements the wide-field illumination source by mesh retessellation of the entire domain, creating an additional meshed medium between the source and the main domain, having the same optical properties of air [$\mu_{a}(\lambda )$ and $\mu_{s}(\lambda )$ equal to $0\;{\mathrm{mm}}^{-1}$, and g and n equal to 1].^12^^,^^13^^,^^19^

Finally, the Monte Carlo HSI framework also takes into account the detection and recording of information regarding the simulated photon packets by placing a 1.2×1.2  mm 2-D detector at the top surface of the mouse cortex domain, coextensive with the illumination field from the source. The choice of locating the detector precisely on the surface area of the domain has the advantage of maximizing the solid angle between the reflected photons and the detector, and thus the geometric detection efficiency of the configuration. This is not fully realistic, as it neglects the fraction of light that would be loss due to the distance between imaged target and detector (as well as the presence of the focusing optics), although such loss would only minimally affect the signal-to-noise ratio (SNR) of the results. Nonetheless, with this configuration, the MC framework does not have to take into account any lens or objective for focusing and collection of light in the simulations.

### 2.2 Data Processing and Analysis

Hyperspectral illumination and imaging of the meshed domain representing the exposed brain cortex are reproduced using the MC framework by simulating photon incidence, diffusion, and reflection in each medium at different wavelengths in the NIR range, from 780 to 900 nm. At each execution of the MC code routine, 30 million ($3\times 10^{7}$) photon packets are launched from the planar source, for each simulated wavelength. This number was chosen after performing convergence analysis. The photons reaching the detector surface after interacting with the domain are then recorded, in particular, the information about their final positions on the detector, their weights when they reached the detector and the partial pathlengths each of them have travelled in each medium. The detector is then divided into 185×185  pixels (6.5-μm pixel size) and the detected photons for each wavelength are binned in these pixels according to their final position. The spatial images at each wavelength are then reconstructed by adding up the weights of all the photons binned in each pixel, in order to create a detected intensity map. A similar approach is used to reconstruct spatial maps of the average total photon pathlengths at each wavelength: these are obtained by summing up the partial pathlengths travelled in each medium by all the binned detected photons in each pixel, weighted by their corresponding weights, and then dividing this sum for the sum of the weights of the detected photons binned in that pixel. These maps provide the spatial distribution of the pathlength that a photon, arriving at a certain pixel, has travelled on average in the domain during a single run of the MC framework and for each wavelength. The reconstructed images at each wavelength are then stacked up to form 3-D spatiospectral datasets, called hyperspectral cubes or hypercubes. The same is done for the reconstructed spatial maps of the average total photon pathlengths to create 3-D average total photon pathlength distribution hypercubes.

For the computational studies reported here, two different brain physiological conditions are simulated, according to the different compositions of each medium of the mouse cortex model: (1) a baseline condition, representing the normal resting state of the brain and (2) an acute hypoxic condition, where cerebral oxygenation and metabolism drop significantly. Therefore, for each condition, the absorption properties of the media constituting the meshed domain of the exposed cortex are determined from their compositions. The scattering properties are only dependent on the selected wavelengths and thus are assumed constant between the two conditions. Water and fat contents are also assumed constant for each medium in both the two conditions. Furthermore, a significant decrease in oxygen saturation, as well as an increase in the total concentration of hemoglobin [to simulate an increase in cerebral blood volume (CBV)], are simulated in the pial vessels and in the subpial gray matter to recreate the hemodynamic response of the exposed cortex during the hypoxic conditions, leading to an overall decrease in the concentration of ${\mathrm{HbO}}_{2}$ and an increase in the concentration of HHb in the whole domain. Similarly, a reduction in the concentration of oxCCO and an increment in the concentration of redCCO are also applied only to the subpial gray matter medium, as to imitate the metabolic response to the lack of oxygen supply in the cerebral cortex. The concentration changes are selected so that the total sum of [oxCCO] and [redCCO] in the entire domain remains constant between the two conditions.^6^

For each simulated condition, image hypercubes and average total photon pathlength hypercubes are reconstructed. Light attenuation changes $\Delta A_{k,l}$ (λ) between the simulated baseline and hypoxia are then calculated for each pixel k, l (for k, l=1…185) and each wavelength λ from the photon intensities $I_{k,l}$ (λ) of the image hypercubes as

$${\Delta A}_{k,l}(\lambda )=-\log_{10}(\frac{I_{k,l,\text{hypoxia}}(\lambda )}{I_{k,l,\text{baseline}}(\lambda )}).$$ (1)

From Eq. (1), hemodynamic and metabolic maps charting the relative changes in concentrations $\Delta [{\mathrm{HbO}}_{2}]$, Δ[HHb], and Δ[oxCCO] of ${\mathrm{HbO}}_{2}$, HHb, and oxCCO, respectively, between the two conditions are estimated: this is done by applying the modified Beer–Lambert’s law (MBLL) to the simulated light attenuation changes $\Delta A_{k,l}$ (λ), pixel by pixel.^6^^,^^25^ Therefore, for each pixel k, l, the following system of algebraic equations is set as

$$[\begin{array}{c} {\Delta A}_{k,l}(\lambda_{1})\\ {\Delta A}_{k,l}(\lambda_{2})\\ \vdots \\ {\Delta A}_{k,l}(\lambda_{M}) \end{array}]=[\begin{array}{ccc} \varepsilon_{{\mathrm{HbO}}_{2}}(\lambda_{1}) & \varepsilon_{\mathrm{HHb}}(\lambda_{1}) & \varepsilon_{\mathrm{diffCCO}}(\lambda_{1})\\ \varepsilon_{{\mathrm{HbO}}_{2}}(\lambda_{2}) & \varepsilon_{\mathrm{HHb}}(\lambda_{2}) & \varepsilon_{\mathrm{diffCCO}}(\lambda_{2})\\ \vdots & \vdots & \vdots \\ \varepsilon_{{\mathrm{HbO}}_{2}}(\lambda_{M}) & \varepsilon_{\mathrm{HHb}}(\lambda_{M}) & \varepsilon_{\mathrm{diffCCO}}(\lambda_{M}) \end{array}]\times [\begin{array}{c} {\mathrm{PL}}_{k,l}(\lambda_{1})\\ {\mathrm{PL}}_{k,l}(\lambda_{2})\\ \vdots \\ {\mathrm{PL}}_{k,l}(\lambda_{M}) \end{array}]\times [\begin{array}{c} \Delta {\left[{\mathrm{HbO}}_{2}\right]}_{k,l}\\ \Delta {\left[\mathrm{HHb}\right]}_{k,l}\\ \Delta {\left[\mathrm{oxCCO}\right]}_{k,l} \end{array}],$$ (2)

where $\varepsilon_{\mathrm{diffCCO}}(\lambda )$ are the oxidized–reduced difference molar extinction coefficients of CCO^6^ (Table 4 in Appendix), ${\mathrm{PL}}_{k,l}$ (λ) are the values, in each pixel, of the mean between the average total photon pathlengths in the baseline and hypoxic conditions obtained from the corresponding hypercubes, whereas M is the total number of wavelengths selected for the specific simulation. The hemodynamic and metabolic maps are finally obtained by solving in all the pixels the corresponding systems of algebraic equations in Eq. (2) for the three unknowns $\Delta [{\mathrm{HbO}}_{2}]$, Δ[HHb], and Δ[oxCCO], using the Moore–Penrose pseudoinverses of the matrices of the molar extinction coefficients.^26^^,^^27^

## 3 Computational Studies

In the first computational study (study 1), feasibility and performances of HSI are assessed by running the MC framework for the maximum allowable number of wavelengths (121) in the range 780 to 900 nm and simulating the two conditions previously described. The capability of HSI to reconstruct correct hemodynamic and metabolic maps is evaluated, in particular regarding image quality, as well as the accuracy in the quantification of the relative changes in the concentrations of ${\mathrm{HbO}}_{2}$, HHb, and oxCCO. In addition, corrections in the algorithm for the analysis of the simulated data are introduced and explored to check if the accuracy in the calculated estimates of $\Delta [{\mathrm{HbO}}_{2}]$, Δ[HHb], and Δ[oxCCO] can be improved, as well as to reduce any cross talk or partial pathlength effects during data postprocessing.

The second study (study 2) with the MC framework is aimed at understanding how the performances of HSI in monitoring hemodynamics and metabolism are influenced by the specific choice of the wavelengths. Different combinations and numbers of wavelengths in the NIR range are tested in order to find an optimal selection of the spectral bands for maximizing precision of the quantitative data.

Cross talk between hemoglobin and CCO and partial pathlength effects are the main targets for the third study (study 3): the MC framework is used to examine the magnitude of the errors introduced by these factors in the reconstructed maps of hemodynamics and metabolism and to verify the physiological origin of the optical signals that are measured from the simulated data. This is done by comparing the realistic scenario tested in study 1 with ideal and hypothetical scenarios, where one or more concentrations of the chromophores remain constant between the two conditions.

The fourth and final study (study 4) explores the implementation of localized hyperspectral illumination and detection on the simulated domain, as a way to identify the best configuration to efficiently apply HSI to the measurement of the hemodynamic and metabolic states of the exposed cortex.

### 3.1 Study on HSI Performances and Accuracy (Study 1)

The first study on the performances and accuracy of HSI in reconstructing quantitative hemodynamic and metabolic maps of brain activity from the exposed cortex domain is conducted using the maximum allowable number of wavelengths in the NIR range from 780 to 900 nm, consisting of 121 wavelengths at 1-nm sampling, for both the baseline and the hypoxic condition. The compositions of the two media for both the simulated conditions are reported in Table 1,^20^ from which the absorption properties used in the simulations are obtained.

**Table 1:** Different compositions of each medium in the meshed domain of the mouse brain cortex, for both the two simulated conditions (baseline and hypoxia).

Medium composition	Baseline condition		Hypoxic condition	
	Gray matter	Vasculature	Gray matter	Vasculature
W (%)	70	50	70	50
F (%)	10	1	10	1
[Hb] (μM)	2325.6	2325.6	3023.3	3023.3
B (%)	3.75	100	3.75	100
S (%)	85	85	50	50
[oxCCO] (μM)	4	0	1	0
[redCCO] (μM)	1	0	4	0
$\mu_{s}$ at 835 nm (${\mathrm{mm}}^{-1}$)	9.1841	9.1841	9.1841	9.1841
$\mu_{a}$ at 835 nm (${\mathrm{mm}}^{-1}$)	0.0275	0.5559	0.0294	0.6492

Note: W, water content; F, fat content; [Hb], concentration of hemoglobin; B, blood content; S, oxygen saturation; [oxCCO], concentration of oxCCO; [redCCO], concentration of redCCO; and an average concentration of hemoglobin in blood equal to 150  g/L is considered, for the baseline.^20^^,^^28^

At the onset of the acute hypoxic condition, an oxygen saturation drop ΔS of −35% is mimicked in the pial vasculature and in the subpial gray matter, compared to the baseline.^29^^,^^30^ Simultaneously, an increase of +30% in the total concentration [Hb] of hemoglobin in the pial vasculature and in the subpial gray matter is also simulated, as to replicate an overall increase in CBV during hypoxia.^31^ These two simulated phenomena correspond to a theoretical increase Δ[HHb] in the concentration of HHb of +1162.79 and +43.60  μM, in the pial vasculature and in the subpial gray matter, respectively, as well as to a theoretical decrease $\Delta [{\mathrm{HbO}}_{2}]$ in the concentration of ${\mathrm{HbO}}_{2}$ equal to −465.12 and −17.44  μM, in the pial vasculature and in the subpial gray matter, respectively. For the metabolic response, it is assumed that the relative concentration change Δ[oxCCO] of oxCCO in the subpial gray matter is equal to −3  μM (this is mirrored by an equivalent increase in [redCCO]).^32^^,^^33^

### 3.2 Study on Optimal Selection of Wavelengths (Study 2)

For the second study, focused on evaluating the influence of the number and selection of NIR wavelengths on the quality and accuracy of the HSI data, the previous simulations for the two conditions (baseline and hypoxia) are repeated by changing the designated wavelengths for the illumination. Specifically, the following combinations of wavelengths are tested: (1) an arbitrary number of wavelengths in the range 780 to 900 nm, consisting of 25 wavelengths at 5-nm sampling and (2) an optimal selection of 8 wavelengths (784, 800, 818, 835, 851, 868, 881, and 894 nm) that was estimated by Arifler et al.^34^ to be an ideal minimum combination of spectral bands for bNIRS to differentiate between the signals of hemoglobin and CCO with <2% mean error, compared to the “gold standard” of 121 wavelengths. The results of the two runs of the MC framework at different wavelengths are then compared with those of study 1, performed at the maximum allowable number of 121 wavelengths. This is intended to demonstrate that changing the number of wavelengths does not significantly affect the results of the quantification of the spatial changes in the concentrations of ${\mathrm{HbO}}_{2}$, HHb, and oxCCO (as long as the selected wavelengths are uniformly sampled in the NIR interval between 780 and 900 nm). Moreover, the outcomes of study 2 also aim at validating that the optimal selection of eight wavelengths for bNIRS is enough to obtain accurate results also in HSI of the exposed cortex with minimal differences from the results with 121 wavelengths.

### 3.3 Assessment and Mitigation of Cross Talk and Partial Pathlength Effects (Study 3)

Evaluation of the presence and severity of cross talk and partial pathlength effects on the simulated hyperspectral data is conducted in the third study. This is done by re-running twice the simulations performed in study 1 while changing the optical properties of the hypoxic condition both times. Specifically: (1) first, simulations with the MC framework are run with only the metabolic response occurring (only the concentrations of redCCO and oxCCO change by ±3  μM, respectively), with no hemodynamic response (the saturation drop ΔS and the increase in [Hb] are equal to zero in the whole domain, thus $\left[{\mathrm{HbO}}_{2}\right]$ and [HHb] do not change between the two conditions). (2) Second, the MC simulations are repeated this time with only the hemodynamic response occurring (the concentrations of ${\mathrm{HbO}}_{2}$ and HHb change according to the drop ΔS in oxygen saturation equal to −35% and the increase in [Hb] equal to +30%), whereas [oxCCO] and [redCCO] remain constant between the two conditions (no metabolic response is simulated). For both sets of simulations, the optimal combination of eight wavelengths tested in study 2 (784, 800, 818, 835, 851, 868, 881, and 894 nm) is selected, for each condition.

The new data from both runs of the MC framework are then compared with the results of study 1 to assess the influence of cross talk and partial pathlength effects in the reconstructed data, as well as to provide an indication of their potential sources. Simulating only the cerebral metabolic response during hypoxia, with no changes in the oxygenation of the tissues, though physiologically unrealistic and improbable, permits to isolate the single optical signature of CCO from those of hemoglobin, thus ideally limiting the occurrence of contamination effects from ${\mathrm{HbO}}_{2}$ and HHb to the minimum. Similarly, the simulation with only the brain hemodynamic response occurring should minimize any cross talk from CCO and related partial pathlength effects. Moreover, this approach can validate the simulated data in the realistic scenario from study 1 by demonstrating that the estimated changes in [oxCCO] are effectively obtained from true changes in the optical properties of the cerebral subpial tissue containing CCO between the two conditions, instead of arising from cross talk signals caused by changes in the concentrations of ${\mathrm{HbO}}_{2}$ and HHb or from the influence of the variance of the photon pathlengths.

### 3.4 Alternative HSI Configuration (Study 4)

**Fig. 2:**
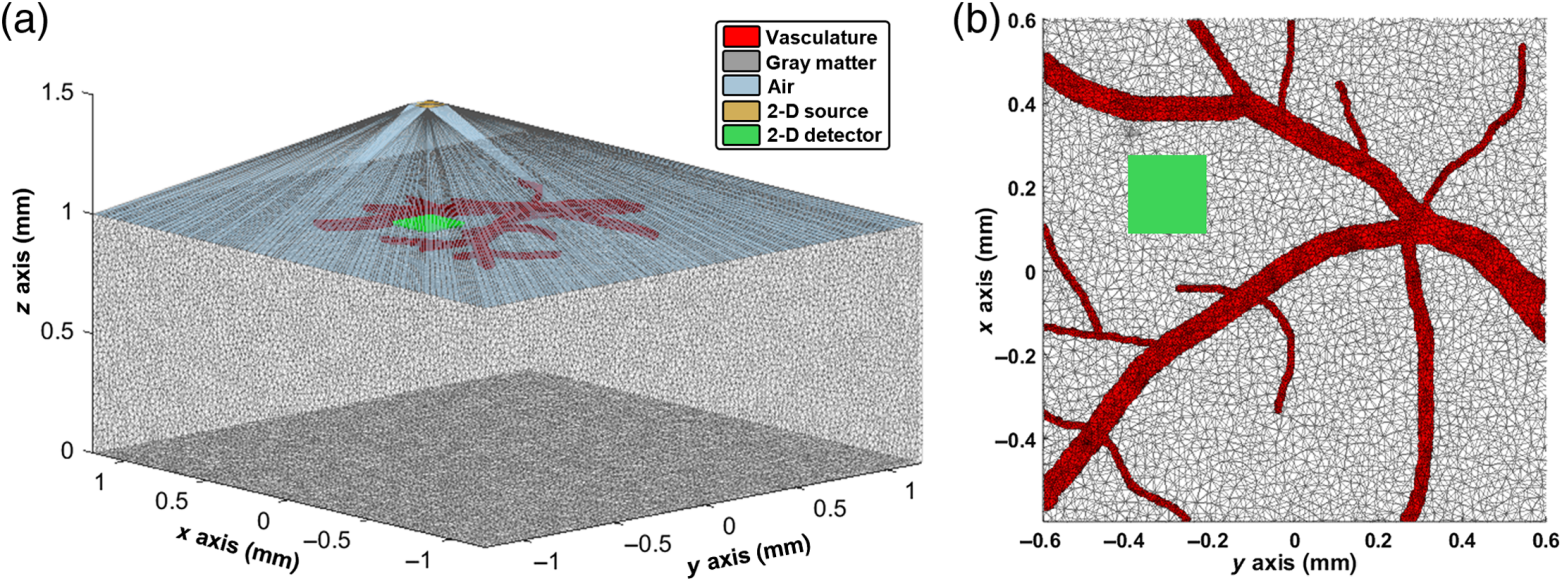
(a) New meshed domain implementing a 2-D source (in gold) and detector (in green) producing a localized 0.2×0.2  mm illumination and FOV. (b) Position of the localized 0.2×0.2  mm FOV of the detector (in green) on the simulated domain, compared to the 1.2×1.2  mm illumination field and detection FOV used in the previous studies.

In the fourth and final study, the Monte Carlo HSI framework is used to explore the implementation of a more localized and selective hyperspectral illumination and detection configuration, designed to improve the accuracy of the quantification of the hemodynamic and metabolic responses in the subpial gray matter, as well as to further mitigate cross talk effects with hemoglobin and partial pathlength effects. In particular, this configuration consists in reducing the illumination area and the FOV of the 2-D detector from 1.2×1.2 to 0.2×0.2  mm (using the same number of pixels, 185×185, in the reconstruction). This is obtained by decreasing the dimension of the source area from 0.6×0.6 to 0.1×0.1  mm and then moving its center to align it to the new detector FOV, as depicted in Fig. 2(a). Such configuration enables to selectively illuminate only a portion of the domain outside the vasculature [Fig. 2(b)], which contains only subpial gray matter, as well as to collect only information from photon packets arriving in the same region. Simulations with the MC framework are run again using the same optical properties used in the study 1 (from Table 1) and with the optimal combinations of eight wavelengths (784, 800, 818, 835, 851, 868, 881, and 894 nm) used in both study 2 and study 3.

## 4 Results

### 4.1 Study 1

**Fig. 3:**
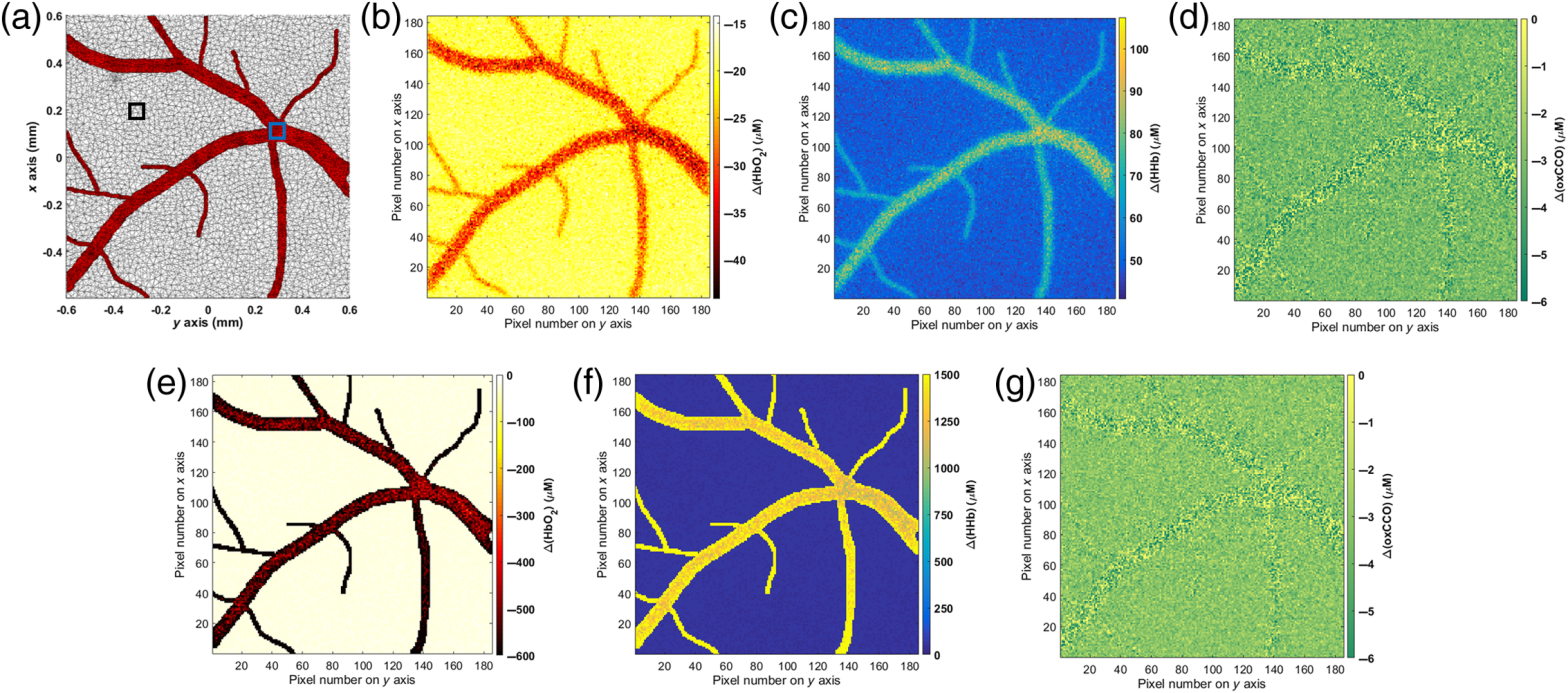
(a) Picture of the 1.2×1.2  mm FOV of the detector on the simulated domain, showing the position of two 65×65  μm ROIs used in the data analysis, one including only pial vasculature (blue square) and the other only subpial gray matter (black square). (b) Hemodynamic map charting the relative changes $\Delta [{\mathrm{HbO}}_{2}]$ in the concentration of ${\mathrm{HbO}}_{2}$ between baseline and hypoxia. (c) Hemodynamic map showing the relative changes Δ[HHb] in the concentration of HHb between baseline and hypoxia. (d) Metabolic map showing the relative changes Δ[oxCCO] in the concentration of oxCCO between baseline and hypoxia. (e) New hemodynamic map of the relative changes $\Delta [{\mathrm{HbO}}_{2}]$ in the concentration of ${\mathrm{HbO}}_{2}$ between baseline and hypoxia, after postprocessing correction. (f) New hemodynamic map of the relative changes Δ[HHb] in the concentration of HHb between baseline and hypoxia, after postprocessing correction. (g) New metabolic map of the relative changes Δ[oxCCO] in the concentration of oxCCO between baseline and hypoxia, after postprocessing correction.

Figure 3 depicts the two hemodynamic maps, for $\Delta [{\mathrm{HbO}}_{2}]$ and Δ[HHb], and the metabolic map of Δ[oxCCO] tracking the relative changes in concentration of the three targeted chromophores during the acute hypoxic condition that was simulated in study 1, using 121 wavelengths between 780 and 900 nm. The hemodynamic maps of the relative changes in concentration of ${\mathrm{HbO}}_{2}$ [Fig. 3(b)] and HHb [Fig. 3(c)] present high image quality and spatial resolution, compared to the actual depiction of the FOV of the simulated domain [Fig. 3(a)]. The large vascular hemodynamic response related to both chromophores is accurately localized within the boundaries of the pial vasculature, resolving both major (about 100  μm in diameter) and minor vessels (about 20  μm in diameter), as well as showing a decrease in the concentration of ${\mathrm{HbO}}_{2}$ and an increase in the concentration of HHb, as theoretically expected. Similarly, a minor hemodynamic response from ${\mathrm{HbO}}_{2}$ and HHb is also reconstructed in the surrounding tissue that is consistent with the simulate changes in oxygen saturation and blood volume in the subpial gray matter. However, from the hemodynamic maps, a large underestimation in the quantification of both $\Delta [{\mathrm{HbO}}_{2}]$ and Δ[HHb] in the pial vasculature clearly emerges. The metabolic map of the relative changes in concentration of oxCCO [Fig. 3(d)] shows a poorer image quality than the hemodynamic maps, due to lower SNR in the processed data for CCO and the presence of spurious measured changes in concentration of CCO in the pial vasculature. These factors make difficult to fully localize the metabolic response and to differentiate between pial vasculature and surrounding tissue with high spatial resolution. Only the major pial vessels (about 100  μm in diameter) are partially resolved in the metabolic map.

Evaluation of the accuracy in quantifying the correct relative changes in the concentrations of ${\mathrm{HbO}}_{2}$, HHb, and oxCCO is performed by calculating and analyzing the spatial averages of the concentration changes $\Delta [{\mathrm{HbO}}_{2}]$, Δ[HHb], and Δ[oxCCO] in specific regions of interest (ROIs) in the hemodynamic and metabolic maps. Two ROIs of 10×10  pixels, both corresponding to square regions of 65×65  μm in the FOV, are selected: (1) one including only pial vasculature and (2) one including only subpial gray matter. The position and size of the two ROIs on the FOV of the detector are shown in Fig. 3(a). The concentrations changes for each chromophore are spatially averaged across the pixels of each ROI. The values of the averages $\left\langle \Delta [{\mathrm{HbO}}_{2}]\right\rangle$, ⟨Δ[HHb]⟩, and ⟨Δ[oxCCO]⟩ in the two ROIs are reported in Table 2 and compared with the corresponding theoretical values. The values of the average concentration changes $\left\langle \Delta [{\mathrm{HbO}}_{2}]\right\rangle$ and ⟨Δ[HHb]⟩ in the ROI associated with the pial vessels reproduce the trend of the expected temporal hemodynamic response to the simulated lack of oxygenation to brain tissue, although they are significantly lower (−32.89  μM for ${\mathrm{HbO}}_{2}$ and 82.78  μM for HHb) than the theoretical simulated changes (−465.12  μM for ${\mathrm{HbO}}_{2}$ and 1162.79  μM for HHb). The corresponding quantification error is equal to about 92.9%. In addition, an erroneous decrease in Δ[oxCCO] is also estimated for the same ROI in the pial vasculature (−2.929  μM), which is in the same order of magnitude of the actual simulated change in the concentration of oxCCO (−3  μM). Finally, the quantification of the relative changes $\Delta [{\mathrm{HbO}}_{2}]$, Δ[HHb], and Δ[oxCCO] in the concentration of ${\mathrm{HbO}}_{2}$, HHb, and oxCCO in the subpial gray matter shows smaller estimation errors of about 10.4%, 11.3%, and 5.2%, respectively, as demonstrated by the value of the average concentration changes $\left\langle \Delta [{\mathrm{HbO}}_{2}]\right\rangle$, ⟨Δ[HHb]⟩, and ⟨Δ[oxCCO]⟩ in the central ROI (−19.32  μM for ${\mathrm{HbO}}_{2}$, 48.54  μM for HHb, and −3.156  μM for oxCCO), which are all close to the theoretical changes in the simulated chromophores (−17.44  μM for ${\mathrm{HbO}}_{2}$, 43.60  μM for HHb, and −3  μM for oxCCO).

**Table 2:** Comparison between the spatial average changes $\left\langle \Delta [{\mathrm{HbO}}_{2}]\right\rangle$, ⟨Δ[HHb]⟩, and ⟨Δ[oxCCO]⟩ in the concentrations of ${\mathrm{HbO}}_{2}$, HHb, and oxCCO, respectively, and the corresponding theoretical values in two different ROIs of 10×10  pixels on the reconstructed hemodynamic and metabolic maps: (1) for the results of study 1, at 121 NIR wavelengths, both before (A) and after correction (B); and (2) for the results of study 2, at 25 NIR wavelengths (C) and at 8 optimal NIR wavelengths (D), both after correction.

Study/dataset	ROI	${\mathrm{HbO}}_{2}$ (μM)		HHb (μM)		oxCCO (μM)	
		Theoretical	$\left\langle \Delta [{\mathrm{HbO}}_{2}]\right\rangle$	Theoretical	⟨Δ[HHb]⟩	Theoretical	⟨Δ[oxCCO]⟩
1/A	Vasculature	−465.12	−32.89±1.238	1162.79	82.78±1.588	0	−2.929±0.632
	Gray matter	−17.44	−19.32±0.330	43.60	48.54±0.680	−3	−3.156±0.203
1/B	Vasculature	−465.12	−474.7±10.57	1162.79	1196.2±20.6	0	−2.929±0.632
	Gray matter	−17.44	−19.39±0.332	43.60	48.72±0.682	−3	−3.167±0.204
2/C	Vasculature	−465.12	−474.2±10.92	1162.79	1192.3±17.7	0	−2.843±0.601
	Gray matter	−17.44	−19.37±0.297	43.60	48.56±0.714	−3	−3.117±0.218
2/D	Vasculature	−465.12	−474.9±13.04	1162.79	1199.9±34.5	0	−3.021±0.831
	Gray matter	−17.44	−19.39±0.373	43.60	48.57±0.867	−3	−3.102±0.299

Both the large underestimation in the changes of concentration of vascular ${\mathrm{HbO}}_{2}$ and HHb in the hemodynamic maps, as well as the occurrence of spurious measured changes in the concentration of oxCCO in the pial vasculature, could be connected to partial pathlength effects. The latter should not be confused with cross talk, because the erroneous measured values of Δ[oxCCO] are not induced by a genuine change in the concentration of this chromophore (since it is not present in the ROI), but they are due to the significant difference between the partial pathlengths of the detected photons that travelled in the pial vasculature and those that travelled in the surrounding subpial brain tissue. This is further investigated and validated by the results of study 3.

**Fig. 4:**
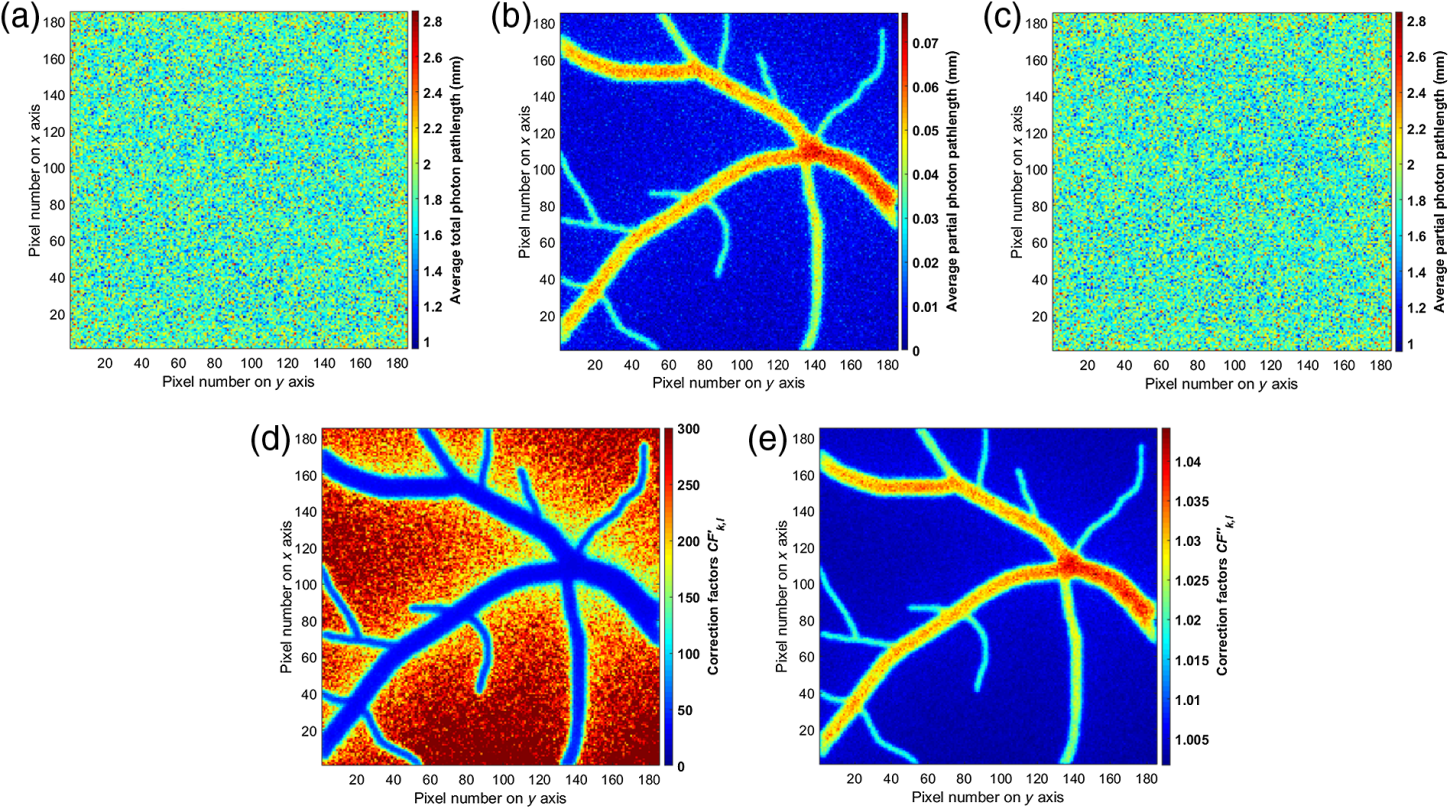
(a) Average total photon pathlength map at 835 nm. (b) Average partial photon pathlength map in the pial vasculature at 835 nm. (c) Average partial photon pathlength map in the subpial gray matter at 835. (d) Map of the correction factors ${\mathrm{CF}}_{k,l}^{\prime }$ obtained from the mean ratios between the average total pathlengths of the detected photons and the average partial pathlengths of the same photons in the pial vasculature, across all the wavelengths and between both simulated conditions. (e) Map of the correction factors ${\mathrm{CF}}_{k,l}^{\prime \prime }$ obtained from the mean ratios between the average total pathlengths of the detected photons and the average partial pathlengths of the same photons in the subpial gray matter, across all the wavelengths and between both simulated conditions.

Figure 4 shows examples, at 835 nm, of the average total pathlength maps of the detected photons across the entire domain [Fig. 4(a)], as well as the average partial pathlength maps of the detected photons in the pial vasculature [Fig. 4(b)] and in the subpial gray matter [Fig. 4(c)], respectively. The maps compare the fractions of the average total pathlengths travelled by the detected photons in each of the two media, during the baseline condition. It can be seen that the partial pathlengths of the detected photons in the pial vasculature are considerably shorter than the partial pathlengths the same photons travelled in the subpial gray matter. The latter also account for more than 97% of the average total pathlength. Moreover, the comparison between the average partial pathlength maps reveals that the majority of photons that were detected in pixels located on the pial vasculature have effectively travelled mostly in the subpial gray matter. This could explain both the significant underestimation of $\Delta [{\mathrm{HbO}}_{2}]$ and Δ[HHb] in the pial vessels, as well as the occurrence of the spurious measured changes Δ[oxCCO] in the same vascular medium, resulting from applying MBLL from Eq. (2) and using the average total pathlength of the detected photons.

A postprocessing correction of the hemodynamic and metabolic maps using the information about the average partial photon pathlengths is here proposed, to primarily improve the quantification of the changes of concentrations of ${\mathrm{HbO}}_{2}$ and HHb in the pial vasculature. Two maps of correction factors, ${\mathrm{CF}}_{k,l}^{\prime }$ and ${\mathrm{CF}}_{k,l}^{\prime \prime }$ (for each pixel k, l), are produced: (1) ${\mathrm{CF}}_{k,l}^{\prime }$ are the means across all the selected M wavelengths (in this case M=121) of the ratios between the average total pathlengths ${\mathrm{PL}}_{k,l}(\lambda )$ travelled by the detected photons and the average partial pathlengths ${\mathrm{PL}}_{k,l,\text{vessel}}(\lambda )$ they travelled in the pial vasculature (for each wavelength λ). (2) ${\mathrm{CF}}_{k,l}^{\prime \prime }$ are the means across all the selected M wavelengths of the ratios between the average total pathlengths ${\mathrm{PL}}_{k,l}(\lambda )$ travelled by the detected photons and the average partial pathlengths ${\mathrm{PL}}_{k,l,\text{gray}}(\lambda )$ they travelled in the subpial gray matter (for each wavelength λ). Thus

$${\mathrm{CF}}_{k,l}^{\prime }=\frac{1}{M}\overset{M}{\underset{i=1}{\sum }}[\frac{{\mathrm{PL}}_{k,l}(\lambda_{i})}{{\mathrm{PL}}_{k,l,\text{vessel}}(\lambda_{i})}]\;{\mathrm{CF}}_{k,l}^{\prime \prime }=\frac{1}{M}\overset{M}{\underset{i=1}{\sum }}[\frac{{\mathrm{PL}}_{k,l}(\lambda_{i})}{{\mathrm{PL}}_{k,l,\text{gray}}(\lambda_{i})}].$$ (3)

The two sets of correction maps can be obtained using Eq. (3) for both the baseline and the hypoxic condition, respectively. The two final sets of correction maps [Fig. 4(d) for ${\mathrm{CF}}_{k,l}^{\prime }$ and Fig. 4(e) for ${\mathrm{CF}}_{k,l}^{\prime \prime }$] to apply to the hemodynamic and metabolic maps are calculated from the values of the mean between the corresponding correction factors of the two conditions (for each pixel k, l), respectively.

The postprocessing correction of the hemodynamic and metabolic maps via the two correction maps in Figs. 4(d) and 4(e) is performed selectively using the segmented binary map of the FOV (utilised during the mesh domain creation, as shown in Fig. 1) as a guide. Thus for pixels k, l corresponding to the pial vasculature medium in the binary mask, the following correction is applied to obtain the corrected values $\Delta [{\mathrm{HbO}}_{2}]*$ and Δ[HHb]* of the changes in concentration of ${\mathrm{HbO}}_{2}$ and HHb in the hemodynamic maps only:

$$\Delta {{[\mathrm{HbO}}_{2}]}_{k,l}^{*}=\Delta {\left[{\mathrm{HbO}}_{2}\right]}_{k,l}{\mathrm{CF}}_{k,l}^{\prime }\;\Delta {\left[\mathrm{HHb}\right]}_{k,l}^{*}=\Delta {\left[\mathrm{HHb}\right]}_{k,l}{\mathrm{CF}}_{k,l}^{\prime }.$$ (4)

The correction in Eq. (4) is not applied to the metabolic map since theoretically no CCO in the pial vasculature is simulated. Contrarily, for pixels k, l corresponding to the subpial gray matter medium in the binary mask, this other correction is applied to both the hemodynamic and the metabolic maps to obtain the corrected values $\Delta [{\mathrm{HbO}}_{2}]*$, Δ[HHb]*, and Δ[oxCCO]* of the changes in concentration of ${\mathrm{HbO}}_{2}$, HHb. and oxCCO:

$$\Delta {{[\mathrm{HbO}}_{2}]}_{k,l}^{*}=\Delta {\left[{\mathrm{HbO}}_{2}\right]}_{k,l}{\mathrm{CF}}_{k,l}^{\prime \prime }\;\Delta {\left[\mathrm{HHb}\right]}_{k,l}^{*}=\Delta {\left[\mathrm{HHb}\right]}_{k,l}{\mathrm{CF}}_{k,l}^{\prime \prime }\;\Delta {\left[\mathrm{oxCCO}\right]}_{k,l}^{*}=\Delta {\left[\mathrm{oxCCO}\right]}_{k,l}{\mathrm{CF}}_{k,l}^{\prime \prime }.$$ (5)

In both sets of Eqs. (4) and (5), ${\mathrm{CF}}_{k,l}^{\prime }$ and ${\mathrm{CF}}_{k,l}^{\prime \prime }$ correspond to the two sets of correction factors (for each pixel k, l) calculated from Eq. (3). This selective correction permits one to weight the hyperspectral data by taking into account the large differences in the average partial photon pathlengths between the two media of the domain.

The new hemodynamic and metabolic maps resulting from the postprocessing correction with Eqs. (3)–(5) are depicted in Figs. 3(e)–3(g) for ${\mathrm{HbO}}_{2}$, HHb, and oxCCO, respectively. A large enhancement in image contrast, as well as in the accuracy in the localization of the hemodynamic response in the pial vasculature, is evident from the new hemodynamic maps, due to the significant improvement in the quantification of $\Delta [{\mathrm{HbO}}_{2}]$ and Δ[HHb] in the pial vessels. Contrarily, the correction in the subpial gray matter produces minimal effects in the hemodynamic and metabolic maps. This is due to the similarity between the average total pathlength and the average partial pathlength of the detected photons in the subpial gray matter, as visible by comparing Figs. 4(a) and 4(c).

The efficacy of the postprocessing correction is further corroborated by the values of the spatial averages $\left\langle \Delta [{\mathrm{HbO}}_{2}]\right\rangle$, ⟨Δ[HHb]⟩, and ⟨Δ[oxCCO]⟩ of the concentration changes of ${\mathrm{HbO}}_{2}$, HHb, and oxCCO in the same two ROIs used for the uncorrected maps. Table 2 shows that the quantified values of $\left\langle \Delta [{\mathrm{HbO}}_{2}]\right\rangle$ and ⟨Δ[HHb]⟩ in the pial vasculature are now much closer to the simulated theoretical values (−474.7  μM for $\left\langle \Delta [{\mathrm{HbO}}_{2}]\right\rangle$ and 1192.2  μM for ⟨Δ[HHb]⟩), with estimation errors of about 2.06% and 2.87%, for ${\mathrm{HbO}}_{2}$ and HHb, respectively. However, the analysis in the ROI localized on the subpial gray matter demonstrates negligible differences (<1%) in the estimates of $\left\langle \Delta [{\mathrm{HbO}}_{2}]\right\rangle$, ⟨Δ[HHb]⟩, and ⟨Δ[oxCCO]⟩ between the corrected and the uncorrected maps, for all the three chromophores. This suggests that the postprocessing correction is only necessary for the pial vasculature in the hemodynamic maps.

**Fig. 5:**
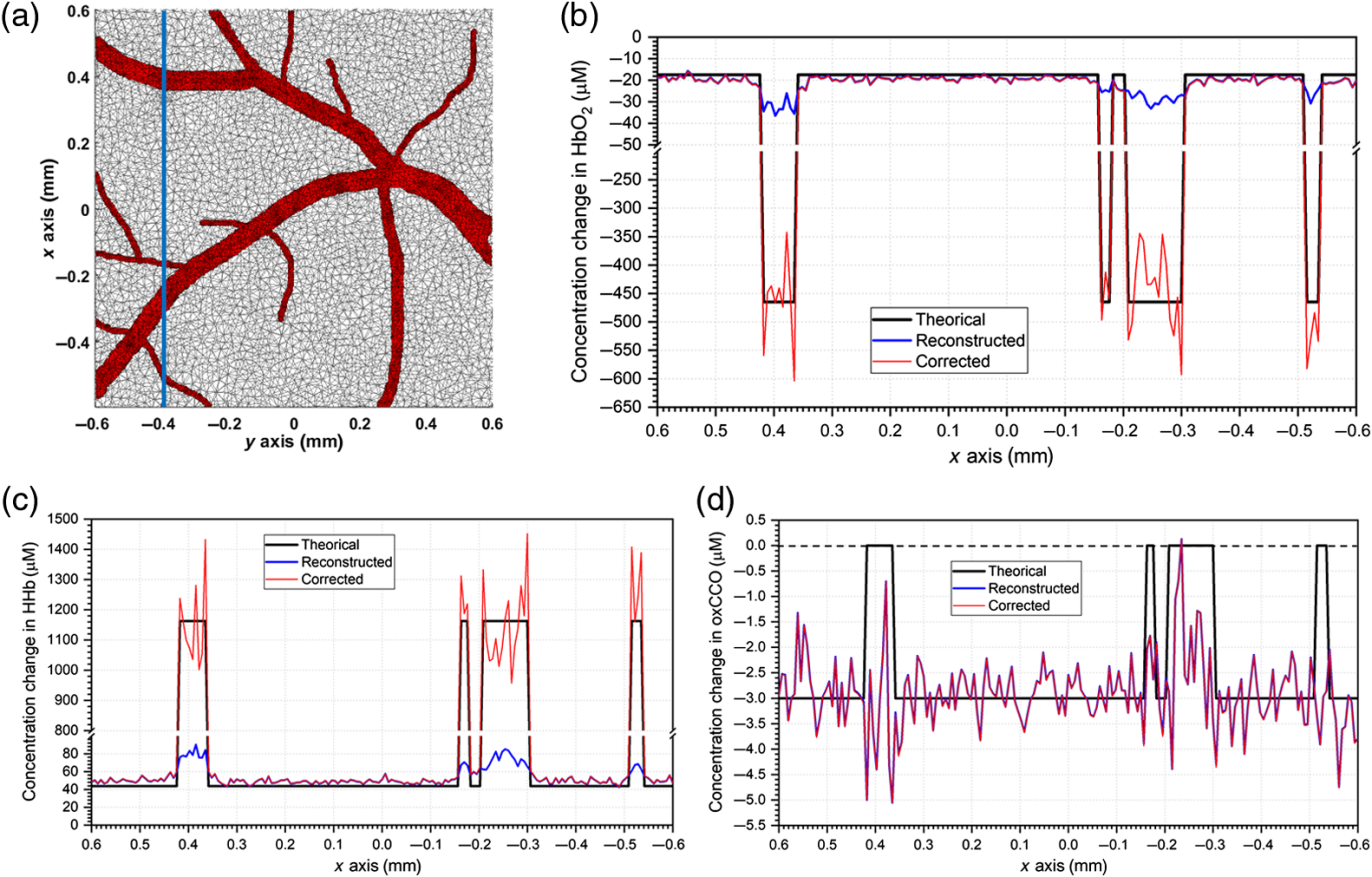
(a) Position on the FOV of the detector of the line of pixel (in blue) used in the data analysis. (b) Relative changes $\Delta [{\mathrm{HbO}}_{2}]$ in the concentration of ${\mathrm{HbO}}_{2}$ along the line of pixels. (c) Relative changes Δ[HHb] in the concentration of HHb along the line of pixels. (d) Relative changes Δ[oxCCO] in the concentration of oxCCO along the line of pixels. Values are depicted both before and after the correction.

A comparison between the cross-section views of the theoretical values of $\Delta [{\mathrm{HbO}}_{2}]$, Δ[HHb], and Δ[oxCCO] and the corresponding reconstructed values, both before correction and after postprocessing correction, is provided in Fig. 5. This analysis on a line of pixels offers additional insight on the partial pathlength effects in all the three maps: in the metabolic map, a large variance characterises the spurious estimated changes in the concentration of oxCCO in the pial vasculature. Figure 5 also further highlights how the postprocessing correction produces: (1) a considerable improvement in spatial localization of the hemodynamic response; (2) a substantial enhancement in the accuracy of the quantification of the relative changes in concentrations of ${\mathrm{HbO}}_{2}$ and HHb; and (3) insignificant differences in the quantification of the relative changes in concentrations of all the three chromophores in the subpial gray matter, in both the hemodynamic and metabolic maps. This last aspect can be clearly seen in Fig. 5(d), where the values of Δ[oxCCO] before and after correction are almost overlapping, as well as for the values of $\Delta [{\mathrm{HbO}}_{2}]$ and Δ[HHb] in the pixels on the subpial gray matter, in both Figs. 5(b) and 5(c).

### 4.2 Study 2

In study 2, similar hemodynamic and metabolic maps for $\Delta [{\mathrm{HbO}}_{2}]$, Δ[HHb] and Δ[oxCCO] are reproduced: (1) first using an arbitrary number of 25 NIR wavelengths between 780 and 900 nm at 5-nm sampling and then (2) using an optimal selection of eight NIR wavelengths (784, 800, 818, 835, 851, 868, 881, and 894 nm),^34^ for the same two simulated brain conditions (baseline and hypoxia). The same postprocessing correction of the hemodynamic and metabolic maps from study 1 is also applied, using Eqs. (3)–(5). Calculation of the spatial averages $\left\langle \Delta [{\mathrm{HbO}}_{2}]\right\rangle$, ⟨Δ[HHb]⟩, and ⟨Δ[oxCCO]⟩ of the relative changes in the concentrations of ${\mathrm{HbO}}_{2}$, HHb, and oxCCO is also performed in the same two ROIs used in study 1, for both the two sets of new maps at 25 and 8 wavelengths. All these values are shown in Table 2: they differ marginally from the corresponding ones in study 1, relative to the data at 121 wavelengths. The differences in the corresponding estimates of $\left\langle \Delta [{\mathrm{HbO}}_{2}]\right\rangle$, ⟨Δ[HHb]⟩, and ⟨Δ[oxCCO]⟩ in both ROIs, between the three combinations of selected wavelengths, varies from 0% to a maximum of 2.1%. In particular, accuracy in quantifying the relative changes in the concentration of ${\mathrm{HbO}}_{2}$, HHb, and oxCCO do not seem to be significantly affected by reducing the spectral information from the maximum allowable number of 121 wavelengths in the selected NIR range down to an optimal combination of only 8. Therefore, these results corroborate the findings of Arifler et al.^34^ that were estimated for bNIRS, extending them also to HSI targeting brain metabolism.

### 4.3 Study 3

**Fig. 6:**
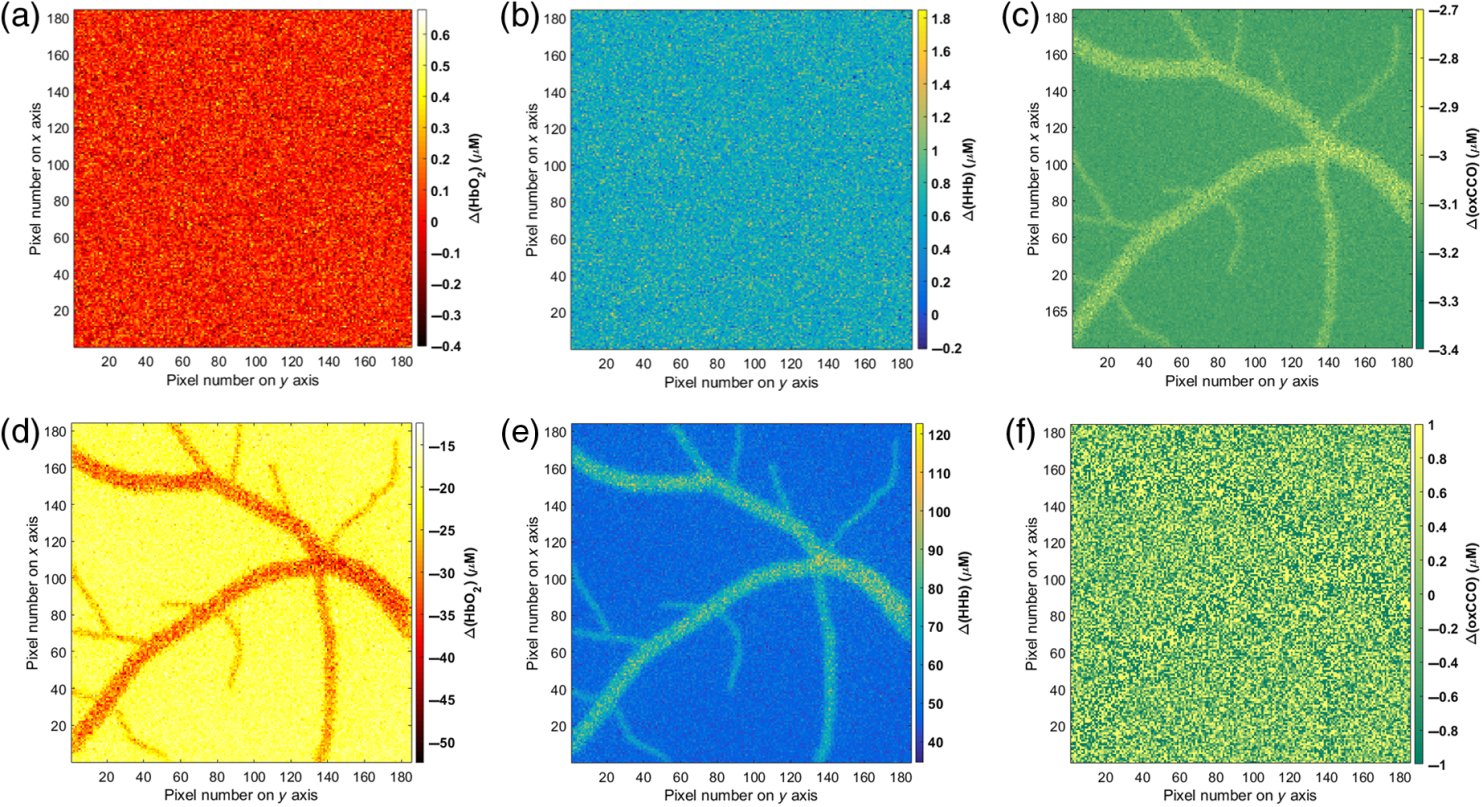
Top row: Hemodynamic and metabolic maps of the relative changes (a) $\Delta [{\mathrm{HbO}}_{2}]$, (b) Δ[HHb], and (c) Δ[oxCCO] in the concentration of ${\mathrm{HbO}}_{2}$, HHb, and oxCCO between baseline and a hypoxic condition, during which only the metabolic response occurs (no simulated changes in hemoglobin). Bottom row: hemodynamic and metabolic maps of the relative changes (d) $\Delta [{\mathrm{HbO}}_{2}]$, (e) Δ[HHb], and (f) Δ[oxCCO] in the concentration of ${\mathrm{HbO}}_{2}$, HHb, and oxCCO between baseline and a hypoxic condition, during which only the hemodynamic response is simulated (no changes in CCO occur).

The results of the first run of the MC framework in study 3, where only the metabolic response is simulated during the hypoxic condition with no changes in hemoglobin in the domain, provide an insight on the influence of cross talk and partial pathlength effects on the reconstruction of the hyperspectral data. The set of hemodynamic and metabolic maps calculated from the hypercubes simulated in this scenario, using the optimal combination of eight wavelengths (784, 800, 818, 835, 851, 868, 881, and 894 nm) from study 2, are shown in the top row of Fig. 6, for $\Delta [{\mathrm{HbO}}_{2}]$, Δ[HHb], and Δ[oxCCO]. No postprocessing correction was performed in this case.

In the absence of any replicated hemodynamic response in the simulations, the hemodynamic maps do not display any contrast provided by the changes $\Delta [{\mathrm{HbO}}_{2}]$ and Δ[HHb] in hemoglobin, neither in the pial vasculature nor in the surrounding tissue, as expected. Therefore, no cross talk effect from CCO is found. The image quality of the metabolic map is higher compared to the results from study 1, due to the lower influence of the optical signatures of hemoglobin in the data. However, nonzero relative changes in the concentration of oxCCO are still estimated in the vessels, even though the pial vasculature does not contain any CCO.

Further validation to these deductions is obtained by looking at the spatial averages $\left\langle \Delta [{\mathrm{HbO}}_{2}]\right\rangle$, ⟨Δ[HHb]⟩, and ⟨Δ[oxCCO]⟩ of the relative changes in the concentrations of ${\mathrm{HbO}}_{2}$, HHb, and oxCCO, again calculated for the same two ROIs analyzed in the previous studies. As reported in Table 3, the values of the averages $\left\langle \Delta [{\mathrm{HbO}}_{2}]\right\rangle$ and ⟨Δ[HHb]⟩ in both ROIs are close to zero. Larger and still non-negligible spurious measurements are estimated for oxCCO from the analysis of the spatial averages ⟨Δ[oxCCO]⟩ in the ROI corresponding to the pial vasculature (−3.018  μM). The magnitude of these is still in the same order of the simulated relative change in concentration of oxCCO (−3  μM). Finally, a more accurate quantification of the relative change Δ[oxCCO] in the metabolic map emerges from the calculation of the spatial average ⟨Δ[oxCCO]⟩ in the ROI corresponding to the gray matter (−3.098  μM), which appears closer to the effective simulated change in oxCCO than the result from study 1 (−3.157  μM). The corresponding estimation error is about 3.27% (against 5.2% in study 1).

**Table 3:** Spatial average changes $\left\langle \Delta [{\mathrm{HbO}}_{2}]\right\rangle$, ⟨Δ[HHb]⟩, and ⟨Δ[oxCCO]⟩ in the concentrations of ${\mathrm{HbO}}_{2}$, HHb, and oxCCO, respectively, in two different ROIs of the reconstructed maps in study 3 (3), obtained for: (A) the simulations with only metabolic response during hypoxia (no correction applied) and (B) the simulations with only hemodynamic response during hypoxia (no correction applied). Both datasets were simulated using eight optimal NIR wavelengths between 780 and 900 nm.

Study/dataset	ROI	${\mathrm{HbO}}_{2}$ (μM)		HHb (μM)		oxCCO (μM)	
		Theoretical	$\left\langle \Delta [{\mathrm{HbO}}_{2}]\right\rangle$	Theoretical	⟨Δ[HHb]⟩	Theoretical	⟨Δ[oxCCO]⟩
3/A	Vasculature	0	0.049±0.026	0	0.628±0.040	0	−3.018±0.025
	Gray matter	0	0.029±0.026	0	0.637±0.071	−3	−3.098±0.022
3/B	Vasculature	−465.12	−32.74±1.336	1162.79	82.17±2.637	0	−0.083±0.798
	Gray matter	−17.44	−19.21±0.356	43.60	47.65±0.832	0	−0.086±0.282

Additional insight on the phenomenon of partial pathlength effect is inferred from the results of the second run of the MC framework in this study: contrarily to the previous run, only the hemodynamic response during the hypoxic condition is simulated in this case, in both the pial vasculature and the subpial gray matter, whereas no change in CCO occurs in the subpial gray matter. Again, the simulations are run for the same optimal combination of eight wavelengths and no postprocessing correction is applied to the three maps.

The bottom row of Fig. 6 illustrates the hemodynamic and metabolic maps of $\Delta [{\mathrm{HbO}}_{2}]$, Δ[HHb], and Δ[oxCCO] obtained from the simulations with only changes in hemoglobin. As expected, the hemodynamic maps, when only changes in hemoglobin occur, again measure and localize the simulated hemodynamic response in both the pial vasculature and the subpial gray matter, similar to the results obtained for study 1 with 121 wavelengths (before postprocessing correction), as seen in Figs. 3(a) and 3(b). The changes $\Delta [{\mathrm{HbO}}_{2}]$ and Δ[HHb] in the pial vessels are still greatly underestimated, suggesting that the underestimation is not affected by the presence of the metabolic response of CCO and thus is not generated by cross talk. Furthermore, no contrast between pial vasculature and subpial gray matter appears in the metabolic map.

The analysis of the spatial averages $\left\langle \Delta [{\mathrm{HbO}}_{2}]\right\rangle$, ⟨Δ[HHb]⟩, and ⟨Δ[oxCCO]⟩ in the two selected ROIs (Table 3) clearly demonstrates the extent of the relative changes in the concentrations of oxCCO still present in the metabolic map is very minimal (−0.086  μM on average in the ROI including only the subpial gray matter), as well as any occurrence of spurious measured changes in the concentration of oxCCO in the pial vessels (⟨Δ[oxCCO]⟩ equal to −0.083  μM in the pial vasculature), compared to the results in study 1 (Table 2).

The findings in study 3 further validate the assumption that the spurious measured signals in a region of the maps are not affected by the presence of the concentration change of another chromophore (cross talk between the chromophores) but purely arise from partial pathlength effects.

### 4.4 Study 4

The new HSI configuration tested in the fourth and final study with the MC framework, implementing and simulating a 0.2×0.2  mm illumination field and detection FOV, explores the possibility to improve accuracy in quantifying brain hemodynamic and metabolic response in the subpial gray matter during the hypoxic condition, compared to the earlier results obtained in study 1 and study 2, without the need of postprocessing correction. The MC framework is run using the optimal combination of eight wavelengths (784, 800, 818, 835, 851, 868, 881, and 894 nm). The reconstruction is performed in the same way as for the previous studies, providing hemodynamic and metabolic maps composed of 185×185  pixels. Since the FOV is now smaller (0.2×0.2  mm, against the previous 1.2×1.2  mm FOV), the size of the pixels decreases from 6.5 to 1.08  μm. No postprocessing correction is applied to the reconstructed hyperspectral data obtained with the new simulated HSI configuration.

The reconstructed maps for the localized illumination and imaging are not reported: this is because they do not show any significant spatial contrast since the new configuration involves the FOV being located entirely over a homogeneous area of subpial gray matter, with no features to be differentiated. Spatial averages $\left\langle \Delta [{\mathrm{HbO}}_{2}]\right\rangle$, ⟨Δ[HHb]⟩, and ⟨Δ[oxCCO]⟩ of the relative changes in the concentrations of ${\mathrm{HbO}}_{2}$, HHb, and oxCCO are calculated from the hemodynamic and metabolic maps for an ROI of 60×60  pixels concentric with the 0.2×0.2-mm FOV. The ROI corresponds to a square region of about 65×65  μm of subpial gray matter. This is done to conduct the spatial average analysis on exactly the same portion of subpial gray matter that was targeted in all the previous studies.

An improvement in the quantification of the concentrations of both ${\mathrm{HbO}}_{2}$ and HHb in the subpial gray matter is achieved with the new configuration, without postprocessing correction, compared to the corresponding values obtained in study 1 for the same ROI (Table 2). The spatial averages $\left\langle \Delta [{\mathrm{HbO}}_{2}]\right\rangle$ and ⟨Δ[HHb]⟩ in the ROI for the new HSI configuration stand at −17.67  μM for $\left\langle \Delta [{\mathrm{HbO}}_{2}]\right\rangle$ and 44.64  μM for ⟨Δ[HHb]⟩, against −19.39 and 48.72  μM, respectively for study 1. The new values are much closer to the theoretical simulated changes in the concentration of ${\mathrm{HbO}}_{2}$ and HHb in the subpial gray matter (−17.44  μM for ${\mathrm{HbO}}_{2}$ and 43.60  μM for HHb). The quantification errors for $\left\langle \Delta [{\mathrm{HbO}}_{2}]\right\rangle$ and ⟨Δ[HHb]⟩ with the new configuration decrease to about 0.75% and 2.11%, respectively, against 10.4% and 11.3% for study 1. This is due to targeting a smaller volume of cerebral tissue, thus reducing the influence of scattering on the estimated average photon pathlengths, as well as to avoiding the illumination of the pial vasculature, which significantly reduces the possibility that a photon may have travelled through that region.

Finally, the quantification of the relative changes in the concentration of oxCCO achieved with the alternative hyperspectral configuration is also more accurate than the one obtained in study 1 (−3.121  μM for ⟨Δ[oxCCO]⟩ with the 0.2×0.2  mm FOV, compared to −3.156  μM with the 1.2×1.2  mm FOV) and thus closer to the simulated metabolic response (−3  μM). The estimation error of ⟨Δ[oxCCO]⟩ with the new HSI configuration is about 4.03% (compared to 5.2% with the larger FOV used in study 1). This is the most accurate quantification of the metabolic response from oxCCO obtained among all the reported studies (excluding the unrealistic cases of study 3).

## 5 Discussion

Preliminary studies with the MC framework proved the suitability of HSI as an optical imaging modality for spatially and quantitatively monitoring the hemodynamic and metabolic response of the exposed cortex to hypoxia: the hemodynamic response was correctly localized in the pial vasculature with high spatial resolution, whereas changes in the concentrations of ${\mathrm{HbO}}_{2}$, HHb, and oxCCO were accurately estimated in the subpial gray matter. The results obtained with the MC framework, regarding the monitoring of hemoglobin oxygenation and blood perfusion on the exposed cortex during hypoxia, are comparable and consistent with previous *in vivo* HSI studies using primarily visible and NIR light.^35^^–^^38^

We speculate that both the underestimation in the quantification of the changes in the concentrations of hemoglobin in the pial vasculature, as well as the occurrence of spurious measured changes in the concentration of oxCCO in the same region (where the MC framework did not simulate such concentration change), are only caused by the large differences in the partial pathlengths of the detected photons between the two media. These differences are not taken into account when applying MBLL since this only consider the total pathlength of the photons. Relevant insights on this phenomenon are highlighted by the findings from the first part of the third study: the hemodynamic maps obtained from simulating only metabolic response during hypoxia demonstrated that negligible relative changes in the concentrations of ${\mathrm{HbO}}_{2}$ and HHb occur in both the pial vessels and the surrounding subpial gray matter in the absence of hemodynamic response. Spurious signals from oxCCO still appear in the pial vasculature, in the same order of magnitude of the relative changes in concentrations of oxCCO due to actual metabolism. Nevertheless, quantification of Δ[oxCCO] in the central subpial tissue was still accurate and closer to the actual change in oxCCO than the results of the study 1. This suggests that partial pathlength effects do not affect significantly the quantification of the metabolic response in the same region and the changes in CCO do not arise as a cross talk from the hemoglobin signals. Thus the measured data obtained for Δ[oxCCO] in the subpial gray matter are primarily connected to the optical signature of CCO, proving the efficacy of HSI to retrieve metabolic signal in the exposed cortex. This conclusion is furtherly supported by the results from the second part of third study, where oppositely only the hemodynamic response in the domain was simulated, showing no changes in Δ[oxCCO] in both the hemodynamic and metabolic maps, as expected.

We then proposed a postprocessing, spatially selective correction taking into account the differences in the partial pathlengths of the detected photons, which enhanced image contrast in the hemodynamic maps and the accuracy of the quantification of the hemodynamic response in the pial vasculature with an estimation error of <3%.

No major differences were found in the outcomes of the second study using different numbers and combinations of wavelengths for hyperspectral illumination, compared to the results of the first study using the maximum allowable number of 121 wavelengths. Thus we showed that reducing the number of simulated wavelengths (as long as they are evenly sampled across in the selected NIR range) down to an optimal combination of only eight does not significantly affect the quality of the hyperspectral data, nor provide significant differences in the accuracy of the quantification of both the hemodynamic and metabolic responses. This is consistent with the results by Arifler et al.^34^ on wavelength optimization for simultaneous monitoring of hemoglobin and CCO via bNIRS.

The findings in study 2 can be advantageous for designing an experimental benchtop HSI system for monitoring hemodynamic and metabolism in the exposed cortex of small animals since reducing the number of necessary wavelengths needed to obtain accurate data decreases complexity and cost of the instrumentation, as well as computational burden to process a smaller volume of hyperspectral data.

Finally, the results of the fourth study provided a preliminary proof of concept for the use of an alternative HSI imaging approach based on localized and selective hyperspectral illumination and detection, to increase the accuracy in the quantification of the hemodynamic and metabolic response in the subpial gray matter. This approach improved the accuracy of the quantification of relative changes in the concentrations of ${\mathrm{HbO}}_{2}$, HHb, and oxCCO in that region (estimation errors of <2.5% for hemoglobin and 4% for CCO) without the need of postprocessing correction.

The new hyperspectral illumination and detection approach, as well as the hyperspectral processing algorithms here reported, can be implemented in a benchtop HSI system and validated under controlled experimental conditions, e.g., using blood and yeast liquid phantoms.^39^ Moreover, the tested HSI configuration could be further explored and developed in the future, e.g., by spatially scanning larger FOVs including both vasculature and gray matter or by applying modulated illumination techniques similar to those used in spatial frequency-domain imaging and structured illumination imaging.^40^^,^^41^

The findings of the four studies reported here can be translated into an experimental setting and could improve the performances of any benchtop NIR HSI system that targets the relative changes of concentration of ${\mathrm{HbO}}_{2}$, HHb, and oxCCO on the exposed cortex, whereas the MC framework can be easily coupled with the instrumentation to aid hyperspectral data acquisition and reconstruction for *in vivo* applications.

Further studies and developments of the MC HSI framework can be explored in the future, which can consist of: (1) refining the simulated domain to include also subpial microvasculature; (2) considering potential differences in the scattering properties between pial vasculature and subpial gray matter; and (3) simulating and investigating additional cerebral physiological conditions besides hypoxia, such as hypercapnia, hyperemia, and other abnormal brain hemodynamic and metabolic responses.

## 6 Conclusion

A MC framework simulating NIR HSI quantitative monitoring of the hemodynamic and metabolic states of the exposed cortex has been here described and tested for a realistic meshed domain, generated from *in vivo* data and replicating mouse cerebral pial vasculature and subpial gray matter. We demonstrated its efficacy for modeling hyperspectral illumination and data acquisition, using up to 121 wavelengths in the NIR range between 780 and 900 nm, as well as for reproducing measurements of the relative changes in the concentrations of ${\mathrm{HbO}}_{2}$, HHb, and oxCCO in the form of hemodynamic and metabolic maps. The MC framework can be also flexibly tuned for different applications, as well as different numbers and ranges of wavelengths, making it a powerful and reusable tool for simulating HSI, due to its ability to reproduce complex meshed domains of various types of tissue from real data.

## 7 Appendix

Table 4 in this section reports the absorption coefficients $\mu_{a,H_{2}O}(\lambda )$ and $\mu_{a,\mathrm{fat}}(\lambda )$ of water and fat, respectively, and the molar extinction coefficients $\varepsilon_{{\mathrm{HbO}}_{2}}(\lambda )$, $\varepsilon_{\mathrm{HHb}}(\lambda )$, $\varepsilon_{\mathrm{oxCCO}}(\lambda )$, and $\varepsilon_{\mathrm{redCCO}}(\lambda )$ of ${\mathrm{HbO}}_{2}$, HHb, oxCCO, and redCCO, respectively, which were used to calculate the absorption properties of the simulated domain, with corresponding references or sources. The oxidized–reduced difference molar extinction coefficients $\varepsilon_{\mathrm{diffCCO}}(\lambda )$ of CCO used in Eq. (2) are also reported in Table 4.

**Table 4:** Values in the NIR range between 780 and 900 nm of: (1) the absorption coefficients $\mu_{a,H_{2}O}$ and $\mu_{a,\text{fat}}$ of water and fat, respectively; (2) the molar extinction coefficients $\varepsilon_{{\mathrm{HbO}}_{2}}$, $\varepsilon_{\mathrm{HHb}}$, $\varepsilon_{\mathrm{oxCCO}}$, and $\varepsilon_{\mathrm{redCCO}}$ of ${\mathrm{HbO}}_{2}$, HHb, oxCCO, and redCCO, respectively; and (3) the oxidized–reduced difference molar extinction coefficients $\varepsilon_{\mathrm{diffCCO}}$ of CCO. References and sources of these values are also provided.

Wavelength (nm)	$\mu_{a,H_{2}O}$ (${\mathrm{cm}}^{-1}$)	$\mu_{a,\text{fat}}$ (${\mathrm{cm}}^{-1}$)	$\varepsilon_{{\mathrm{HbO}}_{2}}$ ($M^{-1}\;{\mathrm{cm}}^{-1}$)	$\varepsilon_{\mathrm{HHb}}$ ($M^{-1}\;{\mathrm{cm}}^{-1}$)	$\varepsilon_{\mathrm{oxCCO}}$ ($M^{-1}\;{\mathrm{cm}}^{-1}$)	$\varepsilon_{\mathrm{redCCO}}$ ($M^{-1}\;{\mathrm{cm}}^{-1}$)	$\varepsilon_{\mathrm{diffCCO}}$ ($M^{-1}\;{\mathrm{cm}}^{-1}$)
	Matcher et al.^22^	van Veen et al.^23^	Matcher et al.^24^	Matcher et al.^24^	Measured by Moody^6^	Measured by Moody^6^	Measured at UCL^6^
780	0.01142	0.00409	735.8251	1104.715	3755.500	1794.484	2049.479
781	0.01127	0.00391	741.9921	1080.047	3764.925	1790.206	2060.684
782	0.01118	0.00378	748.2894	1056.812	3773.988	1786.436	2071.889
783	0.01117	0.00366	754.7170	1036.922	3784.138	1781.724	2084.570
784	0.01099	0.00360	761.1011	1017.031	3791.388	1776.323	2097.382
785	0.01087	0.00357	767.9195	997.2270	3798.275	1770.849	2107.544
786	0.01072	0.00355	774.0865	981.9398	3805.525	1764.759	2117.750
787	0.01055	0.00353	780.6009	966.5658	3814.588	1760.083	2126.566
788	0.01061	0.00349	787.2022	951.2786	3822.200	1754.065	2135.469
789	0.01053	0.00346	793.8903	938.2498	3827.638	1748.591	2142.766
790	0.01036	0.00346	800.3179	926.1764	3835.250	1742.864	2150.062
791	0.01024	0.00347	806.7889	914.0596	3842.500	1737.716	2156.663
792	0.01022	0.00347	813.1730	903.1588	3850.475	1732.243	2163.264
793	0.01012	0.00347	819.4703	893.6912	3855.913	1725.428	2175.729
794	0.01005	0.00352	826.2018	884.1801	3862.438	1720.570	2188.149
795	0.00983	0.00359	832.8031	874.9731	3867.513	1714.154	2200.961
796	0.00974	0.00369	838.9701	867.3729	3872.225	1708.136	2213.642
797	0.00969	0.00379	845.5714	859.7728	3878.025	1702.300	2227.323
798	0.00964	0.00387	852.1726	852.1726	3878.388	1693.818	2241.046
799	0.00951	0.00393	858.6002	845.8754	3887.088	1691.751	2251.209
800	0.00956	0.00403	865.0712	839.6650	3891.075	1685.843	2261.328
801	0.00951	0.00413	871.3685	833.5848	3897.963	1680.333	2266.018
802	0.00936	0.00424	877.7960	828.2864	3900.138	1674.968	2270.709
803	0.00939	0.00434	884.2670	823.5092	3903.038	1669.276	2275.616
804	0.00935	0.00442	890.6946	818.7754	3907.750	1666.703	2280.611
805	0.00932	0.00454	897.0787	814.3890	3912.825	1661.011	2286.430
806	0.00928	0.00467	903.5497	810.9147	3915.363	1655.973	2292.119
807	0.00923	0.00482	909.9772	807.3100	3919.350	1651.224	2298.504
808	0.00929	0.00497	916.1876	803.7922	3920.438	1647.273	2304.931
809	0.00938	0.00511	922.4849	801.1865	3925.513	1643.684	2310.794
810	0.00942	0.00527	928.8690	798.4938	3930.588	1642.451	2316.614
811	0.00952	0.00547	935.1663	795.8881	3933.488	1639.080	2320.609
812	0.00958	0.00567	941.3767	793.8903	3935.300	1636.978	2324.692
813	0.00969	0.00583	947.5437	791.9794	3937.475	1632.736	2327.514
814	0.00964	0.00594	953.6673	790.1988	3937.475	1628.966	2330.294
815	0.00984	0.00603	960.0514	788.5919	3940.375	1625.704	2332.900
816	0.00995	0.00615	966.3487	787.2022	3942.550	1623.746	2335.505
817	0.01011	0.00633	972.5591	785.8993	3943.275	1619.831	2338.198
818	0.01021	0.00654	978.6392	784.6833	3944.000	1616.025	2340.891
819	0.01019	0.00674	984.5456	783.9884	3944.363	1612.038	2343.583
820	0.01047	0.00691	990.8429	783.2067	3944.725	1609.138	2346.319
821	0.01056	0.00708	997.0533	782.5118	3944.363	1604.969	2347.188
822	0.01060	0.00724	1002.960	781.9906	3942.550	1602.359	2348.013
823	0.01093	0.00740	1008.823	781.5129	3939.288	1597.393	2346.493
824	0.01123	0.00758	1015.033	780.9918	3940.013	1596.196	2344.886
825	0.01144	0.00775	1020.853	780.6878	3939.650	1594.819	2342.324
826	0.01218	0.00786	1027.020	780.6009	3938.925	1592.861	2339.718
827	0.01260	0.00796	1033.056	780.3838	3937.113	1590.106	2336.287
828	0.01333	0.00799	1039.050	780.2969	3935.300	1587.786	2332.900
829	0.01384	0.00802	1044.652	780.2100	3933.850	1585.611	2329.425
830	0.01459	0.00803	1050.428	780.2100	3932.038	1585.575	2325.908
831	0.01510	0.00803	1056.117	780.2100	3929.500	1583.074	2322.694
832	0.01586	0.00801	1061.850	780.2100	3927.325	1583.545	2319.393
833	0.01638	0.00794	1067.713	780.2100	3924.425	1579.521	2316.831
834	0.01656	0.00785	1073.619	780.2969	3922.975	1579.920	2314.225
835	0.01699	0.00775	1079.439	780.2969	3921.888	1580.645	2312.531
836	0.01740	0.00764	1084.824	780.3838	3921.525	1581.479	2310.707
837	0.01740	0.00757	1090.427	780.5140	3919.713	1579.739	2310.012
838	0.01758	0.00751	1095.942	780.6878	3913.188	1577.854	2309.231
839	0.01773	0.00739	1101.501	780.9918	3906.663	1576.948	2308.406
840	0.01795	0.00726	1106.930	781.2958	3902.313	1575.389	2307.493
841	0.01813	0.00715	1112.532	781.5129	3899.050	1575.933	2305.626
842	0.01805	0.00703	1117.917	781.8169	3894.700	1575.860	2303.715
843	0.01814	0.00692	1122.999	782.1209	3889.263	1572.271	2301.327
844	0.01822	0.00685	1128.297	782.5118	3887.088	1574.193	2298.894
845	0.01835	0.00678	1133.595	783.1198	3882.738	1572.380	2296.636
846	0.01848	0.00669	1138.807	783.6844	3874.038	1569.951	2294.204
847	0.01856	0.00661	1143.888	784.2924	3868.238	1569.988	2292.814
848	0.01883	0.00651	1149.100	784.8136	3864.975	1571.764	2291.338
849	0.01898	0.00643	1154.398	785.4216	3860.988	1571.619	2290.599
850	0.01913	0.00637	1159.306	785.8993	3854.825	1573.939	2289.905
851	0.01908	0.00633	1164.213	786.8982	3845.763	1569.734	2287.299
852	0.01936	0.00632	1169.121	787.8102	3837.425	1571.764	2284.736
853	0.01931	0.00631	1173.898	788.8091	3830.175	1571.365	2279.916
854	0.01930	0.00633	1178.719	789.8948	3825.463	1572.525	2275.139
855	0.01934	0.00639	1183.887	790.9805	3817.850	1568.646	2267.929
856	0.01959	0.00647	1188.186	792.1097	3809.875	1568.864	2260.720
857	0.01960	0.00656	1193.094	793.2823	3802.988	1568.755	2252.121
858	0.01969	0.00662	1197.697	794.7155	3796.825	1569.843	2243.609
859	0.01972	0.00666	1202.301	796.1052	3787.763	1565.529	2236.747
860	0.01981	0.00674	1206.774	797.4950	3777.613	1563.064	2229.755
861	0.02001	0.00680	1211.074	799.1018	3768.550	1564.478	2225.325
862	0.02002	0.00690	1215.286	800.7087	3760.575	1561.868	2220.852
863	0.02008	0.00705	1219.673	802.3156	3751.513	1558.496	2216.422
864	0.01996	0.00724	1224.189	804.1831	3743.538	1554.146	2212.122
865	0.02034	0.00751	1228.272	806.0940	3736.288	1555.705	2206.346
866	0.02021	0.00786	1232.180	808.0049	3732.300	1561.106	2200.440
867	0.02045	0.00822	1236.393	810.0026	3723.238	1559.765	2193.535
868	0.02044	0.00860	1240.388	812.0004	3711.275	1560.708	2186.629
869	0.02085	0.00899	1244.601	814.0850	3698.950	1559.185	2176.727
870	0.02108	0.00938	1248.683	816.3868	3688.438	1559.584	2166.869
871	0.02116	0.00979	1252.592	818.6885	3679.013	1557.264	2152.364
872	0.02111	0.01022	1256.197	821.0771	3671.038	1554.509	2137.858
873	0.02138	0.01074	1259.801	823.5092	3661.975	1557.373	2125.046
874	0.02160	0.01133	1263.797	825.8978	3654.000	1557.771	2112.278
875	0.02172	0.01198	1267.488	828.2864	3643.488	1557.046	2102.376
876	0.02174	0.01264	1270.659	830.8053	3633.338	1558.315	2092.474
877	0.02217	0.01345	1274.263	833.3677	3621.846	1557.554	2084.049
878	0.02236	0.01430	1277.781	835.8866	3611.733	1559.983	2075.754
879	0.02278	0.01527	1281.082	838.4924	3599.480	1558.931	2067.893
880	0.02282	0.01636	1284.252	840.9678	3588.823	1560.744	2060.076
881	0.02290	0.01752	1287.466	843.5736	3577.005	1561.179	2052.476
882	0.02355	0.01867	1290.376	846.0925	3571.713	1564.043	2044.876
883	0.02366	0.01992	1293.459	848.6983	3560.874	1563.861	2035.365
884	0.02413	0.02121	1296.760	851.3909	3548.839	1564.260	2025.984
885	0.02421	0.02262	1299.670	853.9967	3538.508	1562.774	2015.908
886	0.02437	0.02414	1302.362	856.6024	3526.038	1561.251	2005.789
887	0.02440	0.02581	1305.489	859.1648	3512.879	1562.375	1995.409
888	0.02447	0.02754	1308.052	861.7705	3501.678	1562.085	1984.986
889	0.02504	0.02932	1310.484	864.2895	3489.461	1561.686	1972.001
890	0.02592	0.03116	1313.350	866.7649	3477.173	1564.985	1958.885
891	0.02579	0.03305	1316.173	869.1970	3466.080	1565.529	1946.204
892	0.02573	0.03491	1318.561	871.5856	3456.148	1567.378	1933.523
893	0.02612	0.03674	1320.646	873.9742	3441.575	1566.834	1919.799
894	0.02602	0.03833	1323.165	876.2760	3433.346	1569.154	1906.119
895	0.02643	0.03983	1325.858	878.4909	3423.595	1566.943	1891.135
896	0.0263	0.04121	1327.942	880.4886	3409.603	1567.378	1876.109
897	0.02667	0.04256	1330.070	882.4864	3398.764	1569.371	1861.430
898	0.0274	0.04384	1331.981	884.3539	3386.403	1569.118	1846.750
899	0.02781	0.04506	1333.762	886.0910	3373.099	1568.683	1833.852
900	0.02858	0.04633	1336.150	887.7848	3358.490	1570.386	1820.823
